# Editorial: Oxytocin in brain health and disease: how can it exert such pleiotropic neuromodulatory effects?

**DOI:** 10.3389/fnmol.2023.1215351

**Published:** 2023-05-17

**Authors:** Francesca Talpo, Navjot Kaur, Gerardo Biella

**Affiliations:** ^1^Department of Biology and Biotechnology Lazzaro Spallanzani, University of Pavia, Pavia, Lombardy, Italy; ^2^Department of Neuroscience, School of Medicine, Yale University, New Haven, CT, United States; ^3^Istituto Nazionale di Fisica Nucleare, Pavia, Italy

**Keywords:** oxytocin, oxytocin–therapeutic use, oxytocinergic pathways, oxytocin receptors, pleiotropic neuropeptide, social behavior, neurodegeneration, autism

## Introduction

The pituitary hormone, oxytocin (Oxt), is gaining more and more interest from researchers in neuroscience over the years. Such attention is due to the multitude of heterogeneous effects on neural circuits and pro-social behavioral responses that Oxt elicits through its receptor, the Oxt receptor (OxtR). Imbalances in the oxytocinergic system are implicated in neuropsychiatric diseases associated with altered socio-emotional competence (i.e., autism spectrum disorder, depression), but also in some neurodegenerative diseases, such as Huntington's Disease and amyotrophic lateral sclerosis. Understanding the complex neurobiology of the oxytocinergic system in physiologic and pathologic conditions is a still open scientific challenge. The potentiality of Oxt to be used as a drug treatment further increases the need to extend, collect, and organize knowledge concerning this molecule.

In this special issue, we feature an assortment of contributions (8 reviews, 1 opinion, 2 original research articles) exploring through different approaches and from multiple angles the specific effects exerted by Oxt in the brain. This collection expands and collects current understanding in the neurobiology of Oxt and will provide direction and guidance for future studies in this field.

## Reviews and opinion

Oxt is widely released in the brain, exerting specific neuromodulation of each cerebral region. Manjila et al. integrated brain wide connectivity of Oxt neurons with OxtR expression in mice. Accordingly, they provided insights for three functional circuit-based modules across the whole brain, modulated by the Oxt. These modules regulate respectively (i) the internal state (functional circuits for 1. Attention, 2. Threat, alert, and defense states, 3. Sleep/awake states), (ii) somatic/visceral responses (functional circuits for 1. Pain, 2. Sensory/motor regulation, 3. Body physiology and metabolism), (iii) cognitive responses (functional circuits for 1. Learning and memory, 2. Reward and value assessment, 3. Reproduction). The specific effects of Oxt on the neurons of one of these circuit—the learning and memory circuit, involving the hippocampal formation—have been finely investigated in multiple studies, as review in Talpo et al. Variations in the oxytocinergic functional modulation of the different neurons in each hippocampal subregion appear to account for distinct information processing tasks exerted by each subregion, comprehensively summarized in this review.

An additional element of complexity in the neuromodulation operated by Oxt is due to the presence in many brain regions of higher order G-protein coupled heteroreceptor complexes of the OxtR. Borroto-Escuela et al. reviewed the existence of D2R-OxtR, OxtR-GHS-R1a, 5-HT2AR-OxtR, and 5-HT2CR-OxtR heterocomplexes. In line with this evidence crosstalk between Oxt and other neurotransmitters should be seriously considered.

Central neuromodulatory functions of Oxt are related especially to emotional and social behaviors. Triana-Del Rio et al., Coccia et al., Chen et al. and Muscatelli et al. described different aspects of these pro-social behavioral responses elicited by the Oxt. Triana-Del Rio et al. summarized how Oxt signaling in the limbic network modulates social and stress/threat-related behaviors, starting from the description of the underling cellular and molecular mechanisms. Authors describe how these responses are especially important in the interactions with conspecifics. Coccia et al. extended this concept by explaining the importance of empathy in social decision-making to survive in social environment. Indeed, Oxt modulates some of the major components of social decision-making (emotional discrimination, social recognition, emotional contagion, social dominance, and social memory) and is associated to empathy-like and pro-social behaviors in rodents. In the context of conspecific interactions, strong evidence of the role of Oxt is offered by the analysis of the parental caregiving behaviors of male mice, as discussed in the opinion paper by Chen et al. Referring to a recent paper by Kazunari Miyamichi's team (Inada et al., 2022), authors clarified that plasticity processes occur at hypothalamic oxytocinergic neurons when male mice become fathers, determining a behavioral switch from aggressivity to the conspecific young mice to caring for their pups. Muscatelli et al. also described differences in the dynamics of Oxt related to specific life stages, focusing especially on the role of Oxt in newborn mice. They reported that neonatal Oxt plays a key role in modulating/adapting sensory input and feeding behavior, thus establishing mother-infant bond and structuring all future social interactions.

Impairment of the oxytocinergic system have been reported in many pathological conditions, spanning from autism spectrum disorder to neurodegenerative diseases. Zayan et al. reviewed the modulatory effects elicited by Oxt on the thermosensory system—the sensory system that allows thermoregulation in mammals—that is strongly impaired in autism spectrum disorder. Based on the currently available literature, Bergh et al. described how Oxt could be implicated in Huntington's disease, amyotrophic lateral sclerosis, and frontotemporal dementia. Oxt seems indeed to play a pivotal role in determining altered social cognition and psychiatric features across these diseases. These pieces of evidence open the way for possible therapeutic strategies using Oxt.

## Original research articles

Xu et al. by using medicated lollipops, a well-tolerated modality for an oro-mucosal administration of drug, demonstrated that Oxt blood concentration increased similarly as given by lingual and nasal routes. Accordingly, by taking advantage of an established anti-saccade paradigm, they proved that Oxt modulates top-down social attention by increasing anti-saccade error rates and response latencies for social stimuli and by reducing state anxiety similarly to what obtained following both intranasal (Xu et al., 2019) and lingual (Zhuang et al., 2022) administration. It remains to be evaluated whether these functional effects are mediated in the brain by Oxt crossing the BBB or acting indirectly on the receptor expressed by vagal afferences.

Additionally, Ghafouri-Fard et al. showed that the expression of the oxytocin related genes (FOS, ITPR, RCAN1, and RGS2) was significantly downregulated in periodontitis tissues compared to control tissues, suggesting a potential role for oxytocin-related pathways in the development and progression of periodontitis. However, further research is needed to understand the underlying mechanisms and the correlation between the expression of these genes and the pathological stage of periodontitis.

## Conclusions

The papers in this Research Topic provide insights into the neurobiology of Oxt in health and disease, spanning from the delineation of the pro-social role of Oxt, to the description of the effect of Oxt on specific brain networks, the clarification of crosstalk phenomena between Oxt and other neurotransmitters, the identification of the intracellular pathways activated by Oxt. Then, they provide an overview of the current body of knowledge about Oxt in the brain and highlight the many potentialities of research in this field. In this view, we hope that this Special Topic can stimulate further studies unraveling the missing pieces of knowledge in the mechanisms of oxytocinergic brain modulation, possibly accelerating the development of therapeutic strategies and drugs that use this molecule.

## Author contributions

All authors contributed to this editorial and approved the submitted version.
